# Lassa Fever: Critical Review and Prospects for Control

**DOI:** 10.3390/tropicalmed9080178

**Published:** 2024-08-14

**Authors:** Marianne E. Besson, Michel Pépin, Pierre-Alexandre Metral

**Affiliations:** 1 Department of Public Health, Royal Veterinary College, London NW1 0TU, UK; 2 Department of Virology and Infectiology, VetAgro Sup Lyon University, 69280 Marcy L’Etoile, France; michel.pepin@vetagro-sup.fr; 3 Department of Geography, Grenoble Alpes University, 38400 Grenoble, France; pierre-alexandre.metral@umrpacte.fr

**Keywords:** neglected tropical disease, emerging disease, zoonosis, viral haemorrhagic fever, Lassa Fever, global health

## Abstract

Lassa Fever is a deadly viral haemorrhagic disease, causing annually several hundreds of deaths in West Africa. This zoonotic disease is primarily transmitted to humans by rodents of the genus *Mastomys*, even though other rodents reportedly carry the Lassa virus, while secondary interhuman transmission accounts for approximately 20% of cases. Although this disease has been endemic in rural zones of Nigeria, Sierra Leone, Liberfia, and Guinea for hundreds of years, it is also characterised by epidemic outbreaks in the dry season, responsible for heavy death tolls. No licensed vaccine or satisfying treatment is currently available. Disease management is hindered by the incomplete knowledge of the epidemiology and distribution of the disease, resulting from an inadequate health and surveillance system. Additional scientific constraints such as the genetic diversity of the virus and the lack of understanding of the mechanisms of immune protection complexify the development of a vaccine. The intricate socio-economic context in the affected regions, and the lack of monetary incentive for drug development, allow the disease to persist in some of West Africa’s poorest communities. The increase in the number of reported cases and in the fatality rate, the expansion of the endemic area, as well as the threat Lassa Fever represents internationally should urge the global community to work on the disease control and prevention. The disease control requires collaborative research for medical countermeasures and tailored public health policies. Lassa Fever, created by the interconnection between animals, humans, and ecosystems, and embedded in an intricate social context, should be addressed with a ‘One Health’ approach. This article provides an overview of Lassa Fever, focusing on Nigeria, and discusses the perspectives for the control of disease.

## 1. Lassa Fever: An Overview of the Virology, Clinical Course, and Epidemiology

### 1.1. Understanding Lassa Virus: Genome, Proteins, and Pathogenesis

Lassa Fever (LF) is a zoonotic haemorrhagic fever caused by Lassa virus (LASV), an enveloped single-stranded RNA virus belonging to the *Arenaviridae* family. LASV was first identified in 1969, in the village of Lassa, located in the northeastern Borno State of Nigeria [1].

Arenaviruses have an ambisense segmented genome. It contains two RNA segments, large (L) and small (S) [2]. LASV shows a high genetic variability, that comes mainly from transcription errors, although intrasegmental or intersegmental recombinations may also occur [3,4]. This high genetic diversity, with a nucleotide diversity up to 32% [5] and 7 circulating lineages identified [6], complexifies control measures.

Each segment comprises two genes, oriented in different senses and separated by an intergenic noncoding region. Therefore, each segment encodes one protein in each sense. The large segment encodes the RNA-dependent RNA polymerase and the Z protein, while the small segment encodes the nucleoprotein and the pre-envelop glycoprotein complex [7]. Proteins serve different roles in the infection. Among other functions, the Z protein interact with retinoic acid-inducible gene 1 (RIG-i)-like receptors (RLRs), significantly reducing type I interferon response and therefore suppressing innate immune response [8]. The nucleoprotein masks RNA and binds to the kinase domain of IKK-ε, inhibiting immune response [9].

Immature dendritic cells and macrophages are the initial target cells of LASV [10]. Replication occurs within those cells without either activating the immune response or stimulating T cells. Cytopathic effects (abnormal coagulation, endothelial barrier disruption, and dysfunctional platelet aggregation) and evasion of the immune response contribute to the processes by which viral infection develops and to the pathogenesis of the virus [11,12]. Recent reviews of mechanisms of immune suppression are provided in [13,14].

### 1.2. Lassa Fever: Clinical Course and Mortality Rates

About eighty percent of infections are asymptomatic. In clinical infections, initial symptoms are usually unspecific: high fever, weakness, myalgia, chest and abdominal pain. It is often followed by headaches, sore throat, vomiting and diarrhea. This non-specific clinical picture commonly leads to misdiagnoses, which hinders early diagnosis and patient management [15]. In severe cases, the clinical course evolves to haemorrhages, facial swelling, central nervous system symptoms, multi-organ impairment and hypovolemic shock [16,17,18]. Traditionally, the average case fatality rate (CFR) for symptomatic patients was reported between 15% and 20% [16]. A comparison of confirmed cases to confirmed death by Yaro et al. [19] showed a CFR at 18.5% from 2017 to 2021. Although the CFR depends on the case definition (suspected, probable, or confirmed), the mortality rate has arguably been high over the past years. Considering only the confirmed and probable cases, the CFR in Nigeria was 29.5% from December 2016 to December 2017 [20] and 29.2% in the 2018 epidemics [21], resulting in 191 deaths over the year [22]. An even deadlier epidemic was reported in Liberia over the first half of 2023, with a 30% CFR [23]. Fatality rate can achieve 50% in hospitalised patients [24]. The case fatality per infection is lower, estimated around 1 to 2% [25,26], even if a more thorough assessment of the prevalence of asymptomatic LASV infection is needed.

### 1.3. Medical Interventions and Preventive Measures

Ribavirin is used to treat patients but must be given early in the clinical course [27]. Its mechanism of action is not fully understood. It is hypothesized that ribavirin is an RNA virus mutagen, forcing the virus into what is called an “error catastrophe” [28,29]. Despite its administration, the CFR remains as high as 15% in hospitalised cases [30]. It is also highly toxic [31]. Supportive care remains the primary intervention [13,32]. No vaccine is available, although several vaccine candidates have given promising results in preclinical trials [33]. Four candidates have been advanced into Phase I clinical trials [34], and the first Phase II clinical trial has started in April 2024.

### 1.4. Lassa Fever Epidemiology

Rodents of the species *Mastomys natalensis* (family of *Muridae*) also called multimammate rats, form the primary reservoir of the virus. They were identified through field studies after a large outbreak in Sierra Leone in 1972 [35]. Other rodents have been reported to carry LASV [36]. Zoonotic infection, via indirect or direct contact with rats, accounts for eighty to ninety five percent of the cases [37,38,39]. Hunting of rodents is frequently reported in West Africa, for meat consumption, pest control [40,41], or as a part of community practices and social activities [42,43]. Preparation and consumption of rodent meat is a well-described mode of transmission [44]. Transmission also occurs through exposure to excreta, urine, or other body fluids (saliva, blood): for instance via contaminated food or household items, or by inhalation of rodent excreta [45]. Inadequate sanitation, unhygienic waste disposal [46,47,48] and poor housing quality [49] amplify the risk of exposure to rodents fluids.

Most of LF cases are traditionally reported in the dry season (increase in spillover events) [50,51], although the seasonality of the disease tends to fade, with epidemic outbreaks lasting increasingly longer [52].

A secondary interhuman transmission is possible, mainly via direct contact with blood and body fluids [53].

**Figure 1:**
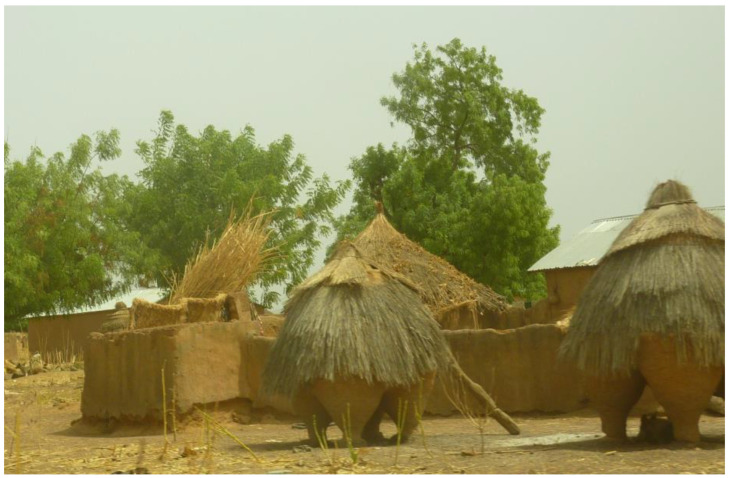
Architectural design and materials of traditional huts and granaries in Northern Nigeria facilitate rodent infestation (original photograph by Marianne Besson).

The multimammate rat is a peri-domestic, commensal species, highly abundant in rural zones. It is well adapted to anthropised territories as it prospers in cultivated lands and inside habitations [54]. It shows a wide distribution across Sub-Saharan Africa. The characteristics of traditional houses and granaries facilitate allow rodents to easily access them (Figure 1). Close contact with rats is a common aspect of daily life in rural areas of West Africa.

## 2. A Devastating Disease in West Africa

LF is both endemic and epidemic in West Africa. Two areas of endemicity have classically been described: the region of the Mano River (Guinea, Sierra Leone, Liberia), and Nigeria. However, it is now acknowledged that the disease exists in other West African countries [39]. LF is estimated to cause between 300,000 and 500,000 cases and 5000 to 6000 deaths annually [55]. However, no precise data can be obtained due to the lack of surveillance, as it mainly affects socio-economically deprived communities in isolated rural zones [56].

The disease burden is especially severe among pregnant women. In a hospital in Sierra Leone, a 30% CFR was observed among 40 women in the third trimester of pregnancy [57]. Abortions are also frequently described: 51 abortions out of 68 women followed by Price et al. [58]. LF can induce unilateral or bilateral sensorineural hearing loss in up to 30% of infected patients [59,60,61]. Because only limited treatment options, if any, are available for deafness in the affected countries, this represents a major liability for social and professional reintegration—even more in countries where deafness is sometimes considered as a sign of intellectual deficiency [62]. It is also strongly suspected that LF can generate depression and psychosis [63,64]. The economic losses associated with the disease –including direct and indirect costs, as well as opportunity costs—have never been evaluated. Mateer and co-authors [61] suggest that deafness only (all causes included) costs 43 million dollars annually to Nigeria.

## 3. An Emerging Disease: Historical and Recent Developments in the Spread of Lassa Fever

LF was first described in the medical literature in the 1920s, under the name “savanna typhus”, as a disease causing prolonged fever, severe headache, neurological signs, hypotension, shock and multi-organ failure, with a high CFR (50%). In the following decades, both sporadic cases and outbreaks (notably among health care workers) were frequently reported in West Africa [65]. Lassa virus was identified in 1969, when a contaminated nurse was repatriated in the USA [1]. However, phylogenetic studies showed that LASV probably originated in Nigeria more than a thousand years ago. The virus subsequently spread to neighboring countries between 300 and 500 years ago, through human migrations during the colonial period [5,66]. Its recent emergence in Mali and the Ivory Coast has been attributed to forced displacements of populations caused by the Sierra Leone civil war (1991–2002) [67].

Since 2016, outbreaks annually hitting Nigeria in the dry season have intensified. In 2020 for instance, 6732 suspected and 1181 confirmed cases were reported nationwide, with 224 deaths [68]. In 2023, the deadliest outbreak in decades was sadly recorded: 9155 suspected cases were reported, 1270 cases were confirmed, with 227 deaths among those. Twenty-eight Nigerian states out of 36 were affected [69]. As of 30 June 2024, 7817 suspected cases and 168 deaths were reported since the beginning of the year in Guinea, Liberia and Nigeria [70]. This surge over the years also reflects an increase in surveillance and diagnosis.

**Figure 2:**
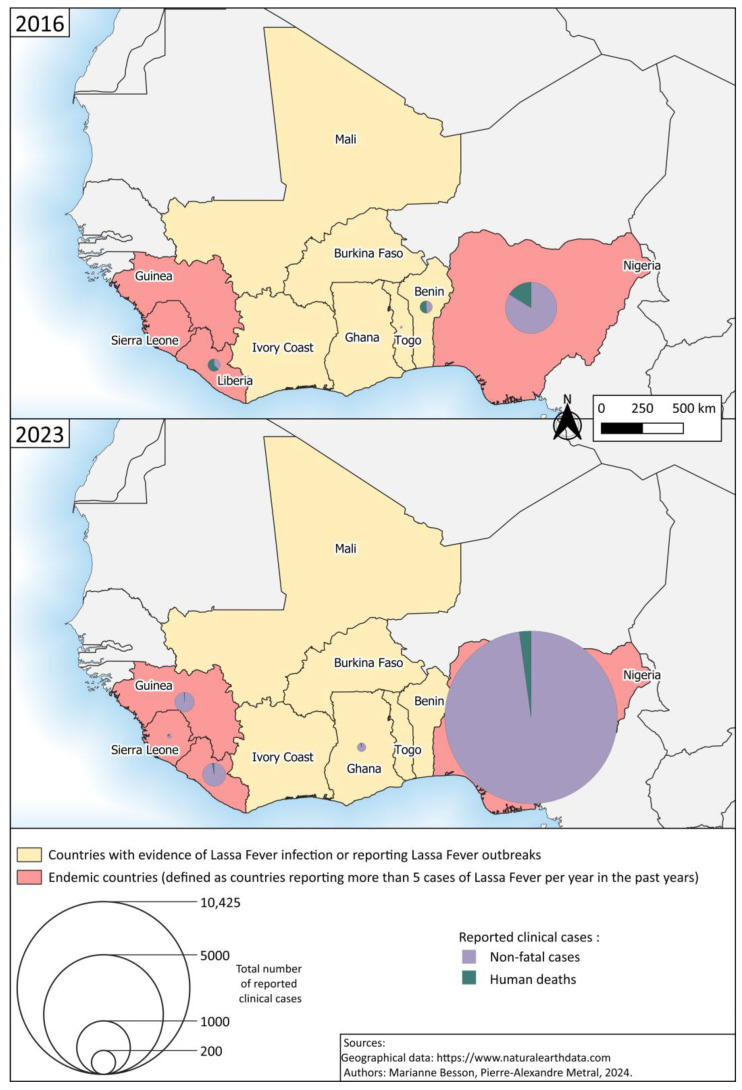
Distribution map showing the numbers of reported clinical cases of LF in 2016 and 2023 in the countries of the endemic zone (estimated numbers only). Based on data from Africa Centres for Disease Control and Prevention [18,77].

Meanwhile, an expansion of the endemic zone has been witnessed in the past decade, with the emergence of new lineages in previously unaffected countries (for instance Ghana, Ivory Coast or Togo) [66,71]. Benin, Burkina Faso, Ghana, Ivory Coast, Mali, Togo are now known as endemic countries, not only because of serological evidence of LF in humans [72], but also because of confirmed autochthonous primary infections [39,73,74] (Figure 2). It is alarming to observe that often, knowledge about the endemic zone is gained when Western travelers contract the disease [75,76].

## 4. Mapping the Spatial Distribution of Lassa Fever

For disease management, it is crucial to map the at-risk areas of Lassa zoonotic transmission. The endemicity area of Lassa Fever might extend beyond the regions where human cases have previously been described. First, LASV circulation in rodents has been evidenced in areas where human cases have not yet been reported [66,78]. The primary reservoir, *Mastomys natalensis*, has the widest distribution of all African rodents, due to its ecological tolerance, adaptability [79,80], and high reproductive capacity. However, the *Mastomys* clade in West Africa is genetically differentiated from the others and could be the only one that carries the virus [81]. LASV has never been isolated east of the border between Nigeria and Cameroon, in a different clade of *M. natalensis* [82]—although LF has been detected in other rodent hosts [83]. Opening ecological corridors (for instance through deforestation) could facilitate the movement of the virus-bearing *Mastomys* populations [84].

Other species have been evidenced to carry the virus, such as *Rattus rattus* and *Mus minutodies* [85,86], as well as species from several other genera of rodents [36,87]. It is still unknown whether the virus can sustain long-term infection in these hosts [88]. As these rodent species occupy different ecological niches, the area of zoonotic infection risk may extend, even more in the context of climate change and increased human mobility [89]. Through modelling of the ecological areas suitable for spillover events, it was estimated than 37.7 million people are at risk of zoonotic infection [78], and this number could drastically increase in the coming years with anthropic changes in West Africa [90]. While non-human primates are susceptible to the infection, LASV and anti-LASV antibodies have also been detected in domestic mammals such as dogs, goats, and pigs, confirming the host plasticity of the virus [91]. These data must urge us to expand research, to fully understand the epidemiological cycle of the virus, the host range, the extent of viral circulation in the reservoir species, and their involvement in the persistence of the virus in wildlife [92]. Active surveillance and surveys of the host species are necessary to prevent and monitor the emergence of the disease.

## 5. Why Lassa Fever Control Measures Are Failing: Challenges to Disease Management and Prevention

### 5.1. Viral Diversity

The inherent characteristics of the virus, including its high genetic diversity, pose significant challenges to the implementation of control measures. To date, seven distinct lineages have been identified [6]. Recent genetic analyses have revealed that selective pressure has driven diversification and local adaptation [93]. However, our understanding of the virus is still incomplete, with several lineages only recently discovered [71,94,95]. This combination of extensive genetic diversity and incomplete characterisation complicates the development of effective medical countermeasures.

Differences in pathogenicity between lineages impact the development of medical countermeasure. Strain Josiah (lineage IV) is the prototypic strain and has been used in many pre-clinical studies, but it is not necessarily the most virulent, as evidenced by challenge in guinea pigs [96]. The vaccines developed targeting this strain could exhibit reduced efficacy against other circulating strains [97].

Even if effective medical countermeasures existed, LF cannot be controlled through one-sided measures, such as massive distributions of prophylactics or drugs. The disease is indeed enrooted in an intricate socioeconomic context. To efficiently combat the emergence of LF, it is imperative to comprehend the political, anthropological, and economic processes that facilitate its existence.

### 5.2. Inadequate Health System

The lack of adequate medical infrastructure and disparities in healthcare accessibility within the affected countries undeniably present significant obstacles to the prevention and control of LF. In Nigeria for instance, above 95% of the population does not have health insurance [98,99]. As an estimated 84 million Nigerians live below the poverty line [100], the financial burden of medical bills is a barrier and only a small proportion of Nigerians benefit from health care [101]. The country presents some of the world worst healthcare indicators [102]. While the urban areas in the South of the country benefit from state-of-the art private hospitals (secondary and tertiary facilities), the vast majority of the rural areas lacks access to medical structure, and primary health care facilities are often decaying, understaffed, deficient in resources [26,101]. The population vulnerability, and the impact of an inadequate, inequitable health network, must be underscored when apprehending the LF issue.

### 5.3. Conflicts and Corruption

Civil unrest, terrorism in the Northeast of Nigeria as well as nationwide fraud and corruption prevent an efficient fight against the disease.

These disastrous observations for Nigeria are unfortunately similar in the other endemic countries: Liberia, Sierra Leone and Guinea respectively rank 178, 181 and 182 out of 191 countries in the world human capital index [103] and suffer from systematic corruption in health sectors [104].

### 5.4. Social Stigma, Mistrust, and Misunderstandings

Richmond and Bagloge [26] also described a fear of social stigma associated with the disease, which consequently dissuades affected individuals from consulting a doctor. Diseases are taboo in many Western African communities, and patients fear rejection from their community. Besides, a defiance towards Western medicine exists [26,105].

Considering that over eighty percent of Lassa Fever cases arise from zoonotic transmission through contact with rodents, sensitization campaigns have been initiated to dissuade communities from hunting and consuming multimammate rats. They do not systematically reach the concerned populations [106], and their messages are often misinterpreted. For instance, through interviews with Sierra Leonean villagers, Bonwitt and co-authors [40] picked up a frequent misidentification of the reservoir (shrews were incriminated instead of the multimammate rats). The general lack of understanding of the attractiveness of rat consumption, and in return a distrust towards public health authorities (reinforced by the bushmeat ban in response to the Ebola crisis) further hindered the campaigns effectiveness [107].

### 5.5. Delayed Diagnosis and Challenges for Healthcare Workers

Limiting secondary transmission is also challenging. The non-descript symptoms frequently result in a wrong identification of the disease by professional health workers. Woyessa and co-authors [108] emphasise that “clinicians and health facilities, especially primary health facilities, need to consider LF as a differential diagnosis when the patient failed to respond to anti-malaria and broad-spectrum antibiotics”. Additionally, endemic zones do not have adequate laboratory capacities, which is a barrier to performing a prompt diagnosis and a confirmation of LASV infection [109].

While rapid diagnostic tests would be an asset, their development is impeded by the high genetic diversity. The pan-Lassa rapid diagnostic test, using a mixture of polyclonal antibodies against LASV recombinant proteins, showed high sensitivity and specificity for patients with a high virus load in a Nigerian specialist hospital setting [110]. It still needs to be tested on more lineages. Distribution to the affected areas remains an issue [111]. Similarly, insufficient supply in ribavirin, which often delays its administration, and limited resources for patient care, hamper cases management [112].

Many non-specialist health facilities also lack personal protective equipment [113]. Instances are also described where, although body protections were available, they were not worn by the health workers, notably when LF diagnosis has not been made yet [15]. The Ebola epidemic in Liberia, Sierra Leone, Guinea led to a heavy death toll among specialist medical forces and weakened capacities for medical response. General practitioners must be sensitised to recognition of communicable diseases so that prophylactic measures can be enforced.

## 6. An Emerging Threat

Several factors contribute to the emergence of the disease. Genetic diversity of the sequenced isolates shows an increase in spillover phenomena [38], especially in the dry season [51]. The multimammate rat forms the dominant rodent species in human-disturbed habitats, in particular after fires [114,115]. Deforestation (notably through slash-and-burn farming) leads to the proliferation of *Mastomys* rats and increases rodent to human contact rates. Expansion of territories suitable for *M. natalensis* also open corridors between geographically distinct populations of rodents, initiating the transmission of LASV in previously disease-free populations [84]. Deforestation, changes in land use and disturbance of ecosystems have consequently been identified as key factors in LF current emergence. Climate change, through an increase in rainfalls in the Gulf of Guinea, is also expected to increase suitability for *M. natalensis* across the region and its reproductive capacity [116]. Models suggest that the number of spillover events could double in the coming decades [81]. Furthermore, mobility and globalisation increase the number of human-to-human transmissions and of exported cases [89].

**Figure 3:**
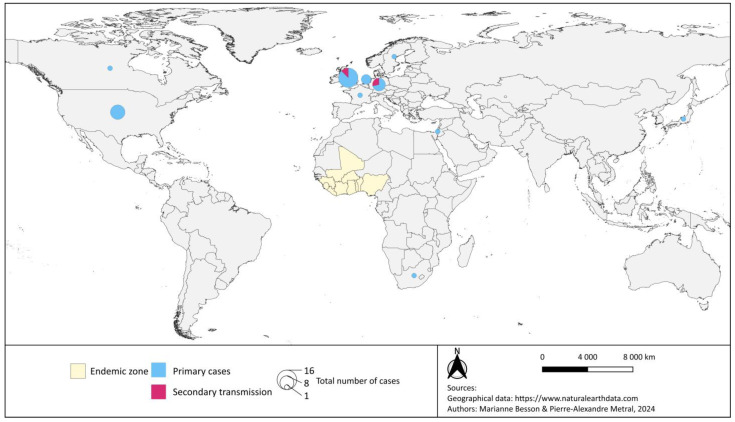
Map of the LF exported cases (primary and secondary transmission) from 1969 to 2024. Based on data from Kofman [122], Choi and Rollin, Wolf et al. [117], World Health Organisation [118].

LF holds the title for most frequently exported viral haemorrhagic disease, with 37 primary exported cases since 1969 (Figure 3) [117,118]. In 2022, three cases and one death were confirmed in the United Kingdom: the first person contracted the disease while traveling in Mali, and transmitted it to two family members upon return to the United Kingdom [118]. The long incubation period (6 to 21 days) [30,119] and the possibility, even low, of an asymptomatic transmission are additional risk factors for secondary propagation in non-endemic countries [120]. Several West African cities are travel hubs, and this interconnexion facilitates the spread of haemorrhagic viral fevers [121].

Even though events presenting a risk of international propagation must be notified to the World Health Organisation (WHO), according to the 2005 International Health Regulations, delays or failures were detected. For instance, in 2016, an outbreak in Benin Republic was not reported, leading to subsequent cases in Togo and in Germany [122,123]. Similarly, in November 2019, two Dutch healthcare professionals were contaminated while performing surgery on an infected patient in Sierra Leone and were repatriated. While the LASV infection was confirmed by RT-PCR on November 20, the Ministry of Health in Sierra Leone formally notified WHO of the ongoing epidemics only a few days later [76,124]. International Health Regulations’ directives for notification of event do not appear in the official Nigerian guidelines for LF case management and infection control [125]. The lack of effective public health surveillance systems in the affected regions and the absence of active surveillance are arguably additional barriers to the disease containment [116].

Nevertheless, because LF is recognised to be a threat for the global community, it received increased international attention over the past decade. It features in the WHO R&D Blueprint list of priority diseases, requiring urgent research and development attention. The international community also aims at improving epidemic preparedness to the disease [126,127]. The Coalition for Epidemic Preparedness Innovations (CEPI) provides increased funding for research efforts [128], and supports programmes for vaccine research or rapid diagnostic tests development [129]. Additionally, CEPI initiated the largest prospective cohort and research capacity building study, the “Enable Lassa Research Programme” following above 23,000 participants in five West African countries (Benin, Guinea, Liberia, Sierra Leone) [130]. This initiative is a key step in strengthening the disease surveillance. It will also help prepare future field trials to assess efficacy and safety of vaccine or therapeutics [131].

## 7. Prospects for Control of Lassa Fever

First, prospects for control dwell in development of both prophylactic and therapeutic medical countermeasures. Because of the lack of profitability of the market in the endemic countries (limited purchasing power of affected communities), there is little financial incentive for pharmaceutical industries to fund research and development initiatives for LF [132]. Therefore, efforts mostly rely on public health institutions and non-governmental organisations [133]. Therapeutic options are currently limited to supportive care and to off-label use of ribavirin, effective only in the early stage of the disease and potentially harmful. New drugs are being investigated for LF management, as reviewed by Garry [134], Melnik [135], and Aloke and co-authors [13]. Favipiravir, integrated in the genome of the virus during replication, is a broad-spectrum inhibitor of viral RNA polymerase [136]. This RNA suppressor successfully treated LASV infection in mice [137] and in macaques [138]. However, the first report of use in two human patients in Togo described nausea and worsening transaminitis, forcing the discontinuation of therapy [139]. Comparatively, the viral entry inhibitor LHF-535 was well tolerated in healthy human volunteers. It showed efficacy in guinea pigs [140], and had an adequate pharmacokinetics profile, but its performance in diseased patients is yet to be evaluated [141]. Immunotherapy offers promising prospects. Interferon-alfacon-1 [142] and other type I interferons [143] could bolster the host innate immune response. Arevirumab-3, a combination of three monoclonal antibodies, protected macaques against parenteral challenge with both lineage II and III LASV isolates [144] and mucosal challenge with an isolate from lineage II [145]. Challenges remain, such as the need for protection against different circulating lineages with various pathogenicity. Similarly, the safety is to be assessed in patients with comorbidities, immunodeficiencies, undernourished or in poor health condition. The first clinical trial is to be set in West Africa, relying on LF specialized treatment centers in Nigeria (such as the Irrua Specialist Teaching Hospital), to identify and efficiently test new drugs candidates [146]. This initiative, launched by a new international consortium of public institutions, INTEGRATE, demonstrates the international will to pro-actively tackle the disease. The question of conveyance and broad availability of drugs in the affected areas, corroded by conflicts, with inadequate and unsafe transport network, remains unsolved. This is even more crucial as treatment must be initiated early in the disease course to be effective, and therefore should be available in primary health care facilities.

Regarding vaccines, four candidates entered the clinical trials, as described in the review by Sulis, Peebles and Basta [147] and by Moore and co-authors [148]: one recombinant measles-vectored LF (MV-LASV) candidate, two candidates using a recombinant vesicular stomatitis virus platform (rVSV-LASV), and one DNA-based candidate. All initiatives are funded by CEPI. MV-LASV candidate includes the genes encoding the nucleoprotein and the glycoprotein precursor from virus strain Josiah. Immune protection conferred in monkey relied on a robust T-cell and humoral response [149,150]. It showed satisfactory safety and immunogenicity in healthy patients in the Phase I human trial [151]. The DNA-based candidate [152], coding the glycoprotein precursor gene, also induced robust T cell responses [153], but failed to meet CEPI’s selection criteria and was discontinued [154]. The rVSV-LASV candidate developed by the International AIDS Vaccine Initiative (IAVI) was also well-tolerated in cohorts from Liberia and from the United States, in the Phase I trial. It elicited robust immune responses, persisting at least one year after vaccination [155]. This candidate has now been advanced into the first-ever Phase II human trial for LF, currently ongoing in Nigeria [156]. Results of Phase I trials for the second rVSV vaccine candidate (developed by Emergent BioSolutions Inc., Gaithersburg, Maryland, USA) [157] and further results for the DNA-based vaccine [152] are to date unpublished.

Because delivery to the affected areas will be complex, several companies are working on reducing the need for cold chain during delivery or storage [158]. However, as highlighted by Leach and Fairfield [159], the challenge is not only to address the “supply-side”, but also to prepare the “demand-side”. How will the population accept these medical measures? Nigeria’s history is marked by instances of vaccine refusal, which can be attributed to several factors, including skepticism towards Western medicine, as well as cultural and religious convictions [160]. In 2003, at the instigation of religious community leaders, five northern states in Nigeria banned the administration of the polio vaccine, claiming that the vaccine was a “Western plot to sterilise women in Nigeria”, or contained human immunodeficiency virus [161].

For a vaccine to be accepted, local perspectives and knowledge should be recognised, and trust should be built with affected communities. The Strategic Advisory Group of Experts (SAGE) on Immunization recommends a multi-component strategy to overcome vaccine hesitancy, including mapping of the factors underlying the hesitancy, impactful communication, and education about vaccines in young individuals. Overall, a collaborative approach should be encouraged rather than top-down directives [162].

Similarly, availability of a drug does not mean that diseased persons will seek medical help. To ensure that the affected population will consult, understanding the relationship of affected communities to health, and fighting the caveats of the health system, will be essential [101]. ‘Healthcare hesitancy’ and the stigma around disease are being more commonly acknowledged, as larger initiatives are now conducted to better characterise the incidence of LF [131]. Understanding local practices will also help limit primary contamination and interhuman transmission.

The ‘One Health’ paradigm reflects the interconnection between human health, animal health and ecosystem health. Above 60% of emerging infectious diseases are of zoonotic origin [163,164], as epitomised by the COVID-19 pandemic. Besides, the ecosystem changes driving LF emergence (deforestation, urbanization, globalization, climate change) potentiate the risk of emergence of many other zoonotic pathogens [165,166,167]. Therefore, recognizing this interrelationship is critical to effectively contend with new health threats [168]. In a broader sense, One Health refers to the understanding and integration of the underlying social, economic, and political dimensions of disease [169]. While policy makers cannot hold sway over the general socio-economic and politic context, some practical solutions have been brought forward, to address the anthropogenic factors that allow the disease emergence and persistence. For instance, rodents thrive in poor sanitary conditions [116]. Sanitation policies, implemented while taking into account the cultural and ethnic specificities [170], are a cost-effective way to prevent LF as well as other scourges [171]. Rather than sectoral interventions and divided research or policy efforts, an integrated approach for surveillance and response, that may address multiple diseases, must be promoted [169].

LF control will be hard to achieve without general economic and sanitary development of the region and reinforcement of health systems. Sufficient resources and supplies in primary care centers, as well as appropriate training of first medical responders, are key to curb the disease progression. Political structures which often underlie zoonotic disease burden must be challenged [172]. Political commitment must be obtained for a sustained effort to achieve LF control. Regional and national leadership is key in the fight against LF, with crucial actors such as the Nigeria Centre for Disease Control, or the Ministries of Health of affected countries. Regional capacity-building for research is also essential [173]. For instance, the Economic Community of West African States (ECOWAS) Regulators and Ethics Committees project aims at empowering regional stakeholders in research initiatives and clinical trials.

At last, Cunningham and co-authors [169] recommend shifting the narrative, from a focus on outbreak control, to addressing LF endemicity. Indeed, despite an increased international awareness in the past decade, LF remains a neglected tropical disease, which receives only limited funding and attention [48].

## 8. Conclusions

Lassa Fever, a deadly zoonotic viral haemorrhagic fever, exemplifies the intricate web of interactions between the health of animals, humans, and ecosystems. As the number of spillover events drastically increased in the past years, the disease poses a multifaceted challenge. It is both a devastating endemic burden in West Africa, and an emerging threat to the global community. Lassa Fever’s emergence and persistence are driven by various ecological and anthropologic factors. The poverty and deep vulnerabilities of the affected population, the inequity in access to healthcare, the lack of sanitation and economic development in the afflicted regions, represent key factors hindering the disease control and prevention. In an endemic region ravaged by conflict and social unrest, a comprehensive, intersectional approach, that considers the complexity of the socio-economic context is essential. International cooperation for research should be reinforced to bridge knowledge gaps (ecology and transmission of the virus, genetic diversity and lineage distribution, accurate mapping of at-risk areas, correlate of immune protections) and to develop effective medical countermeasures. Active and passive surveillance must be strengthened for a better understanding of the epidemiology of the disease. To allow a quick diagnosis, more research institutions are needed in the endemic region, and alternative methods (rapid diagnostic test) should be validated. Because LF is a neglected tropical disease and receives only limited funding, public health interventions ought to be guided by appropriate modelling to target the at-risk populations. Recent advancements, such as the enhancement of the surveillance system and the progress in vaccine development, provide promising prospects for control. A collaborative and sustained effort, led by scientific research, brings hope that the impact of Lassa Fever could be lessened in the West Africa, and its global threat managed. Recognizing the multifaceted nature of Lassa Fever will enable the development of comprehensive strategies that not only tackle the disease but also reduce the risk of other emerging zoonotic diseases.

## Data Availability

The data supporting the creation of the maps derive from literature and can be made available by the authors on request.

## References

[1] Frame J.D., Baldwin J.M., Gocke D.J., Troup J.M. (1970). Lassa Fever, a New Virus Disease of Man from West Africa. Am. J. Trop. Med. Hyg..

[2] Happi A.N., Happi C.T., Schoepp R.J. (2019). Lassa fever diagnostics: Past, present, and future. Curr. Opin. Virol..

[3] Emonet S., Lemasson J.J., Gonzalez J.P., de Lamballerie X., Charrel R.N. (2006). Phylogeny and evolution of old world arenaviruses. Virology.

[4] Zapata J.C., Salvato M.S. (2013). Arenavirus Variations Due to Host-Specific Adaptation. Viruses.

[5] Andersen K.G., Shapiro B.J., Matranga C.B., Sealfon R., Lin A.E., Moses L.M., Folarin O.A., Goba A., Odia I., Ehiane P.E. (2015). Clinical Sequencing Uncovers Origins and Evolution of Lassa Virus. Cell.

[6] Ibukun F.I. (2020). Inter-Lineage Variation of Lassa Virus Glycoprotein Epitopes: A Challenge to Lassa Virus Vaccine Development. Viruses.

[7] Ryu W.S., Ryu W.S. (2017). Chapter 16—Other Negative-Strand RNA Viruses. Molecular Virology of Human Pathogenic Viruses.

[8] Xing J., Ly H., Liang Y. (2015). The Z proteins of pathogenic but not nonpathogenic arenaviruses inhibit RIG-I-like receptor-dependent interferon production. J. Virol..

[9] Zinzula L., Tramontano E. (2013). Strategies of highly pathogenic RNA viruses to block dsRNA detection by RIG-I-like receptors: Hide, mask, hit. Antiviral Res. Antivir. Res..

[10] Baize S., Kaplon J., Faure C., Pannetier D., Georges-Courbot M.C., Deubel V. (2004). Lassa virus infection of human dendritic cells and macrophages is productive but fails to activate cells. J. Immunol..

[11] Russier M., Pannetier D., Baize S. (2012). Immune Responses and Lassa Virus Infection. Viruses.

[12] Schaeffer J., Carnec X., Reynard S., Mateo M., Picard C., Pietrosemoli N., Dillies M.-A., Baize S. (2018). Lassa virus activates myeloid dendritic cells but suppresses their ability to stimulate T cells. PLoS Pathog..

[13] Aloke C., Obasi N.A., Aja P.M., Emelike C.U., Egwu C.O., Jeje O., Edeogu C.O., Onisuru O.O., Orji O.U., Achilonu I. (2023). Combating Lassa Fever in West African Sub-Region: Progress, Challenges, and Future Perspectives. Viruses.

[14] Raabe V., Mehta A.K., Evans J.D., Beitscher A., Bhadelia N., Brett-Major D., Cieslak T.J., Davey R.T., Frank M.G., Iwen P. (2022). Lassa Virus Infection: A Summary for Clinicians. Int. J. Infect. Dis..

[15] Dan-Nwafor C.C., Ipadeola O., Smout E., Ilori E., Adeyemo A., Umeokonkwo C., Nwidi D., Nwachukwu W., Ukponu W., Omabe E. (2019). A cluster of nosocomial Lassa fever cases in a tertiary health facility in Nigeria: Description and lessons learned, 2018. Int. J. Infect. Dis..

[16] Marrama E. (2016). Rapid Risk Assessment: Lassa fever in Nigeria, Benin, Togo, Germany and USA, 24 March 2016. https://www.ecdc.europa.eu/en/publications-data/rapid-risk-assessment-lassa-fever-nigeria-benin-togo-germany-and-usa-24-march.

[17] Basler C.F. (2017). Molecular pathogenesis of viral hemorrhagic fever. Semin. Immunopathol..

[18] Africa Centres for Disease Control and Prevention (2018). Lassa Fever. Africa CDC. https://africacdc.org/disease/lassa-fever/.

[19] Yaro C.A., Kogi E., Opara K.N., Batiha G.E.-S., Baty R.S., Albrakati A., Altalbawy F.M.A., Etuh I.U., Oni J.P. (2021). Infection pattern, case fatality rate and spread of Lassa virus in Nigeria. BMC Infect. Dis..

[20] Nigeria Centre for Disease Control (2017). Lassa Fever Situation Report, Epidemiological Week 46. 24 November–1 December 2017; Report No.: 46. https://ncdc.gov.ng/diseases/sitreps/?cat=5&name=An%20update%20of%20Lassa%20fever%20outbreak%20in%20Nigeria.

[21] Grace J.U.A., Egoh I.J., Udensi N. (2021). Epidemiological trends of Lassa fever in Nigeria from 2015–2021: A review. Ther. Adv. Infect. Dis..

[22] Nigeria Centre for Disease Control (2018). Lassa Fever Situation Report, Epidemiological Week 52. 24 December–31 December 2018; Report No.: 52. https://ncdc.gov.ng/diseases/sitreps/?cat=5&name=An%20update%20of%20Lassa%20fever%20outbreak%20in%20Nigeria.

[23] UK Health Security Agency (UKHSA), Emerging Infections and Zoonoses (EIZ) Team (2023). Infectious Disease Surveillance and Monitoring for Animal and Human Health: Summary January to June 2023. https://www.gov.uk/government/publications/emerging-infections-monthly-summaries/infectious-disease-surveillance-and-monitoring-for-animal-and-human-health-summary-january-to-june-2023.

[24] European Centre for Disease Prevention and Control (2019). Lassa Fever in the Netherlands ex Sierra Leone. https://www.ecdc.europa.eu/sites/default/files/documents/RRA-Lassa-fever-in-the-Netherlands-ex-Sierra-Leone_0.pdf.

[25] McCormick J.B. (1987). Epidemiology and control of Lassa fever. Curr. Top Microbiol. Immunol..

[26] Richmond J.K., Baglole D.J. (2003). Lassa fever: Epidemiology, clinical features, and social consequences. BMJ.

[27] Eberhardt K.A., Mischlinger J., Jordan S., Groger M., Günther S., Ramharter M. (2019). Ribavirin for the treatment of Lassa fever: A systematic review and meta-analysis. Int. J. Infect. Dis..

[28] Crotty S., Maag D., Arnold J.J., Zhong W., Lau J.Y.N., Hong Z., Andino R., Cameron C.E. (2000). The broad-spectrum antiviral ribonucleoside ribavirin is an RNA virus mutagen. Nat. Med..

[29] Crotty S., Cameron C.E., Andino R. (2001). RNA virus error catastrophe: Direct molecular test by using ribavirin. Proc. Natl. Acad. Sci. USA.

[30] World Health Organisation (2017). Lassa Fever, Fact Sheets. https://www.who.int/news-room/fact-sheets/detail/lassa-fever.

[31] Bannister B. (2010). Viral haemorrhagic fevers imported into non-endemic countries: Risk assessment and management. Br. Med Bull..

[32] Alli A., Ortiz J.F., Fabara S.P., Patel A., Halan T. (2021). Management of Lassa Fever: A Current Update. Cureus.

[33] Warner B.M., Safronetz D., Stein D.R. (2018). Current research for a vaccine against Lassa hemorrhagic fever virus. Drug Des. Dev. Ther..

[34] Isaac A.B., Karolina W., Temitope A.A., Anuska R., Joanne E., Deborah A. (2022). Prospects of Lassa fever candidate vaccines. Afr. J. Infect. Dis..

[35] Monath T.P., Newhouse V.F., Kemp G.E., Setzer H.W., Cacciapuoti A. (1974). Lassa Virus Isolation from *Mastomys natalensis* Rodents during an Epidemic in Sierra Leone. Science.

[36] Olayemi A., Oyeyiola A., Obadare A., Igbokwe J., Adesina A.S., Onwe F., Ukwaja K.N., Ajayi N.A., Rieger T., Günther S. (2018). Widespread arenavirus occurrence and seroprevalence in small mammals, Nigeria. Parasites Vectors.

[37] Oloniniyi O.K., Unigwe U.S., Okada S., Kimura M., Koyano S., Miyazaki Y., Iroezindu M.O., Ajayi N.A., Chukwubike C.M., Chika-Igwenyi N.M. (2018). Genetic characterization of Lassa virus strains isolated from 2012 to 2016 in southeastern Nigeria. PLoS Neglected Trop. Dis..

[38] Kafetzopoulou L.E., Pullan S.T., Lemey P., Suchard M.A., Ehichioya D.U., Pahlmann M., Thielebein A., Hinzmann J., Oestereich L., Wozniak D.M. (2019). Metagenomic sequencing at the epicenter of the Nigeria 2018 Lassa fever outbreak. Science.

[39] World Health Organisation (2023). Lassa Fever—Nigeria. WHO Disease Outbreak News. https://www.who.int/emergencies/disease-outbreak-news/item/2023-DON463.

[40] Bonwitt J., Kelly A.H., Ansumana R., Agbla S., Sahr F., Saez A.M., Borchert M., Kock R., Fichet-Calvet E. (2016). Rat-atouille: A Mixed Method Study to Characterize Rodent Hunting and Consumption in the Context of Lassa Fever. Ecohealth.

[41] Ter Meulen J., Lukashevich I., Sidibe K., Inapogui A., Marx M., Dorlemann A., Yansane M.L., Koulemou K., Chang-Claude J., Schmitz H. (1996). Hunting of peridomestic rodents and consumption of their meat as possible risk factors for rodent-to-human transmission of Lassa virus in the Republic of Guinea. Am. J. Trop. Med. Hyg..

[42] Douno M., Asampong E., Magassouba N., Fichet-Calvet E., Almudena M.S. (2021). Hunting and consumption of rodents by children in the Lassa fever endemic area of Faranah, Guinea. PLoS Negl. Trop Dis..

[43] Terashima H., Hewlett B.S. (2016). Social Learning and Innovation in Contemporary Hunter-Gatherers: Evolutionary and Ethnographic Perspectives.

[44] Kernéis S., Koivogui L., Magassouba N., Koulemou K., Lewis R., Aplogan A., Grais R.F., Guerin P.J., Fichet-Calvet E. (2009). Prevalence and Risk Factors of Lassa Seropositivity in Inhabitants of the Forest Region of Guinea: A Cross-Sectional Study. PLoS Negl. Trop Dis..

[45] Pfau C.J. (1996). Arenaviruses. Medical Microbiology.

[46] Abdullahi I.N., Anka A.U., Ghamba P.E., Onukegbe N.B., Amadu D.O., Salami M.O. (2020). Need for preventive and control measures for Lassa fever through the One Health strategic approach. Proc. Singap. Health.

[47] Cadmus S., Taiwo O.J., Akinseye V., Cadmus E., Famokun G., Fagbemi S., Ansumana R., Omoluabi A., Ayinmode A., Oluwayelu D. (2023). Ecological correlates and predictors of Lassa fever incidence in Ondo State, Nigeria 2017–2021: An emerging urban trend. Sci. Rep..

[48] Dalhat M.M., Olayinka A., Meremikwu M.M., Dan-Nwafor C., Iniobong A., Ntoimo L.F., Onoh I., Mba S., Ohonsi C., Arinze C. (2022). Epidemiological trends of Lassa fever in Nigeria, 2018–2021. PLoS ONE.

[49] Bonner P.C., Schmidt W.-P., Belmain S.R., Borchert M., Oshin B., Baglole D. (2007). Poor Housing Quality Increases Risk of Rodent Infestation and Lassa Fever in Refugee Camps of Sierra Leone. Am. J. Trop. Med. Hyg..

[50] Malik S., Bora J., Dhasmana A., Kishore S., Nag S., Preetam S., Uniyal P., Slama P., Mukherjee N., Haque S. (2023). An update on current understanding of the epidemiology and management of the re-emerging endemic Lassa fever outbreaks. Int. J. Surg. Lond. Engl..

[51] McKendrick J.Q., Tennant W.S.D., Tildesley M.J. (2023). Modelling seasonality of Lassa fever incidences and vector dynamics in Nigeria. PLoS Negl Trop Dis..

[52] Nigeria Centre for Disease Control Lassa Fever Situation Report, Epidemiological Week 29. 17 July–23 July 2023; Report No.: 29. https://ncdc.gov.ng/diseases/sitreps/?cat=5&name=An%20update%20of%20Lassa%20fever%20outbreak%20in%20Nigeria.

[53] Fisher-Hoch S.P., Tomori O., Nasidi A., Perez-Oronoz I.G., Fakile Y., Hutwagner L., McCormick J.B. (1995). Review of cases of nosocomial Lassa fever in Nigeria: The high price of poor medical practice. BMJ.

[54] du Plessis J., Russo I., Child M., Child M.F., Roxburgh L., Do Linh San E. (2016). A conservation assessment of *Mastomys* spp.. The Red List of Mammals of South Africa, Swaziland and Lesotho.

[55] Schmaljohn C., Safronetz D. (2019). Editorial overview: Lassa virus. Curr. Opin. Virol..

[56] Simons D. (2022). Lassa fever cases suffer from severe underreporting based on reported fatalities. Int. Health.

[57] Shaffer J.G., Grant D.S., Schieffelin J.S., Boisen M.L., Goba A., Hartnett J.N., Levy D.C., Yenni R.E., Moses L.M., Fullah M. (2014). Lassa Fever in Post-Conflict Sierra Leone. PLoS Negl. Trop. Dis..

[58] Price M.E., Fisher-Hoch S.P., Craven R.B., McCormick J.B. (1988). A prospective study of maternal and fetal outcome in acute Lassa fever infection during pregnancy. BMJ.

[59] Cummins D., McCormick J.B., Bennett D., Samba J.A., Farrar B., Machin S.J., Fisher-Hoch S.P. (1990). Acute Sensorineural Deafness in Lassa Fever. J. Am. Med. Assoc..

[60] Liao B.S., Byl F.M., Adour K.K. (1992). Audiometric comparison of Lassa fever hearing loss and idiopathic sudden hearing loss: Evidence for viral cause. Otolaryngol. Head Neck Surg..

[61] Mateer E.J., Huang C., Shehu N.Y., Paessler S. (2018). Lassa fever-induced sensorineural hearing loss: A neglected public health and social burden. PLoS Negl. Trop Dis..

[62] McPherson B., Brouillette R. (2008). Audiology in Developing Countries.

[63] Macher A.M., Wolfe M.S. (2006). Historical Lassa Fever Reports and 30-year Clinical Update. Emerg. Infect. Dis..

[64] Okogbenin E.O., Obagaye M.O., Aweh B.E., Eriyo W.O., Okogbenin S.A., Okokhere P.O. (2020). One-Year Retrospective Review of Psychiatric Consultations in Lassa Fever, Southern Nigeria. Emerg. Infect. Dis..

[65] Monath T.P. (1975). Lassa fever: Review of epidemiology and epizootiology. Bull. World Health Organ..

[66] Manning J.T., Forrester N., Paessler S. (2015). Lassa virus isolates from Mali and the Ivory Coast represent an emerging fifth lineage. Front. Microbiol..

[67] Lalis A., Leblois R., Lecompte E., Denys C., ter Meulen J., Wirth T. (2012). The Impact of Human Conflict on the Genetics of Mastomys natalensis and Lassa Virus in West Africa. PLoS ONE.

[68] Nigeria Centre for Disease Control (2021). Lassa Fever Situation Report, Epidemiological Week 52. 27 December 2020–2 January 2021; Report No.: 52. https://ncdc.gov.ng/diseases/sitreps/?cat=5&name=An%20update%20of%20Lassa%20fever%20outbreak%20in%20Nigeria.

[69] Nigeria Centre for Disease Control (2023). Lassa Fever Situation Report, Epidemiological Week 52; Report No.: 52. https://ncdc.gov.ng/diseases/sitreps/?cat=5&name=An%20update%20of%20Lassa%20fever%20outbreak%20in%20Nigeria.

[70] Africa Centres for Disease Control and Prevention (2024). Africa CDC Weekly Event Based Surveillance Report, June 2024. https://africacdc.org/download/africa-cdc-weekly-event-based-surveillance-report-june-2024/.

[71] Whitmer S.L., Strecker T., Cadar D., Dienes H.P., Faber K., Patel K., Brown S.M., Davis W.G., Klena J.D., Rollin P.E. (2018). New Lineage of Lassa Virus, Togo, 2016. Emerg. Infect. Dis..

[72] Emmerich P., Thome-Bolduan C., Drosten C., Gunther S., Ban E., Sawinsky I., Schmitz H. (2006). Reverse ELISA for IgG and IgM antibodies to detect Lassa virus infections in Africa. J. Clin. Virol..

[73] Dzotsi E.K., Ohene S.-A., Asiedu-Bekoe F., Amankwa J., Sarkodie B., Adjabeng M., Thouphique A.M., Ofei A., Oduro J., Atitogo D. (2012). The First Cases of Lassa Fever in Ghana. Ghana Med. J..

[74] Centres for Disease Control and Prevention (2014). Outbreak Distribution Map|Lassa Fever|CDC. https://www.cdc.gov/lassa-fever/about/index.html.

[75] Atkin S., Anaraki S., Gothard P., Walsh A., Brown D., Gopal R., Hand J., Morgan D. (2009). The first case of Lassa fever imported from Mali to the United Kingdom, February 2009. Eurosurveillance.

[76] World Health Organisation Regional Office for Africa (2019). Weekly Bulletin on Outbreak and other Emergencies: Week 47: 18–24 November 2019. https://iris.who.int/handle/10665/329974.

[77] Africa Centres for Disease Control and Prevention Africa CDC Weekly Event Based Surveillance Report, 17 February 2024. https://africacdc.org/download/africa-cdc-weekly-event-based-surveillance-report-february-2023/.

[78] Mylne A.Q.N., Pigott D.M., Longbottom J., Shearer F., Duda K.A., Messina J.P., Weiss D.J., Moyes C.L., Golding N., Hay S.I. (2015). Mapping the zoonotic niche of Lassa fever in Africa. Trans. R. Soc. Trop. Med. Hyg..

[79] Colangelo P., Verheyen E., Leirs H., Tatard C., Denys C., Dobigny G., Duplantier J.-M., Brouat C., Granjon L., Lecompte E. (2013). A mitochondrial phylogeographic scenario for the most widespread African rodent, *Mastomys natalensis*. Biol. J. Linn. Soc..

[80] Granjon L. (2016). *Mastomys natalensis*. *IUCN Red List Threat Species*. https://www.iucnredlist.org/species/12868/115107375.

[81] Redding D.W., Moses L.M., Cunningham A.A., Wood J., Jones K.E. (2016). Environmental-Mechanistic Modelling of the Impact of Global Change on Human Zoonotic 2 Disease Emergence: A Case Study of Lassa Fever. Macro-Mechanistic Modelling of Zoonotic Disease Emergence. https://www.repository.cam.ac.uk/handle/1810/256060.

[82] Gibb R., Moses L.M., Redding D.W., Jones K.E. (2017). Understanding the cryptic nature of Lassa fever in West Africa. Ann. Trop. Med. Parasitol..

[83] Happi A.N., Olumade T.J., Ogunsanya O.A., Sijuwola A.E., Ogunleye S.C., Oguzie J.U., Nwofoke C., Ugwu C.A., Okoro S.J., Otuh P.I. (2022). Increased Prevalence of Lassa Fever Virus-Positive Rodents and Diversity of Infected Species Found during Human Lassa Fever Epidemics in Nigeria. Microbiol. Spectr..

[84] Adetola O.O., Adebisi M.A. (2019). Impacts of Deforestation on the Spread of Mastomys natalensis in Nigeria. World Sci. News.

[85] Wulff H., Fabiyi A., Monath T.P. (1975). Recent isolations of Lassa virus from Nigerian rodents. Bull. World Health Organ..

[86] Demby A.H., Inapogui A., Kargbo K., Koninga J., Kourouma K., Kanu J., Coulibaly M., Wagoner K.D., Ksiazek T.G., Peters C. (2001). Lassa Fever in Guinea: II. Distribution and Prevalence of Lassa Virus Infection in Small Mammals. Vector-Borne Zoonotic Dis..

[87] Fichet-Calvet E., Becker-Ziaja B., Koivogui L., Günther S. (2014). Lassa Serology in Natural Populations of Rodents and Horizontal Transmission. Vector-Borne Zoonotic Dis..

[88] Gryseels S., Baird S.J.E., Borremans B., Makundi R., Leirs H., de Bellocq J.G. (2017). When Viruses Don’t Go Viral: The Importance of Host Phylogeographic Structure in the Spatial Spread of Arenaviruses. PLoS Pathog..

[89] Lalis A., Wirth T., Grandcolas P., Maurel M.C. (2018). 11—Mice and Men: An Evolutionary History of Lassa Fever. Biodiversity and Evolution.

[90] Klitting R., Kafetzopoulou L.E., Thiery W., Dudas G., Gryseels S., Kotamarthi A., Vrancken B., Gangavarapu K., Momoh M., Sandi J.D. (2022). Predicting the evolution of the Lassa virus endemic area and population at risk over the next decades. Nat. Commun..

[91] Happi A.N., Ogunsanya O.A., Ayinla A.O., Sijuwola A.E., Saibu F.M., Akano K., Nwofoke C., Elias O.T., Achonduh-Atijegbe O., Daodu R.O. (2024). Lassa virus in novel hosts: Insights into the epidemiology of lassa virus infections in southern Nigeria. Emerg. Microbes Infect..

[92] Arruda L.B., Free H.B., Simons D., Ansumana R., Elton L., Haider N., Honeyborne I., Asogun D., McHugh T.D., Ntoumi F. (2023). Current sampling and sequencing biases of Lassa mammarenavirus limit inference from phylogeography and molecular epidemiology in Lassa fever endemic regions. PLoS Glob. Public Health.

[93] Li Y. (2023). Genetic basis underlying Lassa fever endemics in the Mano River region, West Africa. Virology.

[94] Olayemi A., Cadar D., Magassouba N., Obadare A., Kourouma F., Oyeyiola A., Fasogbon S., Igbokwe J., Rieger T., Bockholt S. (2016). New Hosts of The Lassa Virus. Sci. Rep..

[95] Ehichioya D.U., Dellicour S., Pahlmann M., Rieger T., Oestereich L., Becker-Ziaja B., Cadar D., Ighodalo Y., Olokor T., Omomoh E. (2019). Phylogeography of Lassa Virus in Nigeria. J. Virol..

[96] Clegg J.C., Lloyd G. (1987). Vaccinia recombinant expressing Lassa-virus internal nucleocapsid protein protects guineapigs against Lassa fever. Lancet.

[97] Hallam H.J., Hallam S., Rodriguez S.E., Barrett A.D., Beasley D.W., Chua A., Ksiazek T.G., Milligan G.N., Sathiyamoorthy V., Reece L.M. (2018). Baseline mapping of Lassa fever virology, epidemiology and vaccine research and development. NPJ Vaccines.

[98] Akokuwebe M.E., Idemudia E.S. (2022). A Comparative Cross-Sectional Study of the Prevalence and Determinants of Health Insurance Coverage in Nigeria and South Africa: A Multi-Country Analysis of Demographic Health Surveys. Int. J. Environ. Res. Public Health.

[99] Sasu (2022). Nigeria: Health Insurance Coverage, by Area and Gender|Statista. https://www.statista.com/statistics/1124757/health-insurance-coverage-in-nigeria-by-area-and-gender/.

[100] World Bank (2023). Nigeria Overview: Development News, Research, Data. https://www.worldbank.org/en/country/nigeria/overview.

[101] Okpani A.I., Abimbola S. (2015). Operationalizing universal health coverage in Nigeria through social health insurance. Niger. Med J..

[102] Eke C. (2023). Nigeria—Healthcare. Official Website of the USA International Trade Association. https://www.trade.gov/country-commercial-guides/nigeria-healthcare.

[103] United Nations Development Programme (2022). HDR21-22_Statistical_Annex_HDI_Table.xlsx. https://view.officeapps.live.com/op/view.aspx?src=https%3A%2F%2Fhdr.undp.org%2Fsites%2Fdefault%2Ffiles%2F2021-22_HDR%2FHDR21-22_Statistical_Annex_HDI_Table.xlsx&wdOrigin=BROWSELINK.

[104] Onwujekwe O., Agwu P., Orjiakor C., McKee M., Hutchinson E., Mbachu C., Odii A., Ogbozor P., Obi U., Ichoku H. (2019). Corruption in Anglophone West Africa health systems: A systematic review of its different variants and the factors that sustain them. Heal. Policy Plan..

[105] Ogueji I.A., Ogunsola O.O., Abdalla N.M., Helmy M. (2024). Mistrust of the Nigerian health system and its practical implications: Qualitative insights from professionals and non-professionals in the Nigerian health system. J. Public Health.

[106] Wogu J.O. (2018). Mass media awareness campaign and the prevention of the spread of Lassa fever in the rural communities of Ebonyi State, Nigeria: Impact evaluation. J. Public Health Afr..

[107] Bonwitt J., Dawson M., Kandeh M., Ansumana R., Sahr F., Brown H., Kelly A.H. (2018). Unintended consequences of the ‘bushmeat ban’ in West Africa during the 2013–2016 Ebola virus disease epidemic. Soc. Sci. Med..

[108] Woyessa A.B., Maximore L., Keller D., Dogba J., Pajibo M., Johnson K., Saydee E., Monday J., Tuopileyi R., Mahmoud N. (2019). Lesson learned from the investigation and response of Lassa fever outbreak, Margibi County, Liberia, 2018: Case report. BMC Infect. Dis..

[109] Schroeder L.F., Amukele T. (2014). Medical Laboratories in Sub-Saharan Africa That Meet International Quality Standards. Am. J. Clin. Pathol..

[110] Boisen M.L., Uyigue E., Aiyepada J., Siddle K.J., Oestereich L., Nelson D.K.S., Bush D.J., Rowland M.M., Heinrich M.L., Eromon P. (2020). Field evaluation of a Pan-Lassa rapid diagnostic test during the 2018 Nigerian Lassa fever outbreak. Sci. Rep..

[111] Mazzola L.T., Kelly-Cirino C. (2019). Diagnostics for Lassa fever virus: A genetically diverse pathogen found in low-resource settings. BMJ Glob. Health.

[112] Akpede G.O., Asogun D.A., Okogbenin S.A., Dawodu S.O., Momoh M.O., Dongo A.E., Ike C., Tobin E., Akpede N., Ogbaini-Emovon E. (2019). Caseload and Case Fatality of Lassa Fever in Nigeria, 2001–2018: A Specialist Center’s Experience and Its Implications. Front. Public Health.

[113] Ijarotimi I.T., Ilesanmi O.S., Aderinwale A., Abiodun-Adewusi O., Okon I.M. (2018). Knowledge of Lassa fever and use of infection prevention and control facilities among health care workers during Lassa fever outbreak in Ondo State, Nigeria. Pan. Afr. Med. J..

[114] Avenant N.L., Watson J.P., Schulze E. (2008). Correlating small mammal community characteristics and habitat integrity in the Caledon Nature Reserve, South Africa. Mammalia.

[115] MacFadyen D.N., Avenant N.L., van der Merwe M., Bredenkamp G.J. (2012). The Influence of Fire on Rodent Abundance at the N’washitshumbe Enclosure Site, Kruger National Park, South Africa. Afr. Zool..

[116] Balogun O.O., Akande O.W., Hamer D.H. (2021). Lassa Fever: An Evolving Emergency in West Africa. Am. J. Trop. Med. Hyg..

[117] Wolf T., Ellwanger R., Goetsch U., Wetzstein N., Gottschalk R. (2020). Fifty years of imported Lassa fever: A systematic review of primary and secondary cases. J. Travel Med..

[118] World Health Organisation (2022). Lassa Fever—United Kingdom of Great Britain and Northern Ireland. Disease Outbreak News. https://www.who.int/emergencies/disease-outbreak-news/item/lassa-fever-united-kingdom-of-great-britain-and-northern-ireland.

[119] Grant D.S., Samuels R.J., Garry R.F., Schieffelin J.S. (2023). Lassa Fever Natural History and Clinical Management. Curr. Top Microbiol. Immunol..

[120] Brosh-Nissimov T. (2016). Lassa fever: Another threat from West Africa. Disaster Mil. Med..

[121] Tuite A.R., Watts A.G., Kraemer M.U.G., Khan K., Bogoch I.I. (2019). Potential for Seasonal Lassa Fever Case Exportation from Nigeria. Am. J. Trop. Med. Hyg..

[122] World Health Organisation (2016). Lassa Fever—Germany. Disease Outbreak News. https://www.who.int/emergencies/disease-outbreak-news/item/23-march-2016-lassa-fever-germany-en.

[123] World Health Organisation (2016). Lassa Fever—Togo. Disease Outbreak News. https://www.who.int/emergencies/disease-outbreak-news/item/23-march-2016-lassa-fever-togo-en.

[124] Njuguna C., Vandi M., Liyosi E., Githuku J., Wurie A., Njeru I., Raftery P., Amuzu C., Maruta A., Musoke R. (2022). A challenging response to a Lassa fever outbreak in a non endemic area of Sierra Leone in 2019 with export of cases to The Netherlands. Int. J. Infect. Dis..

[125] Ihekweazu C. (2018). National Guidelines for Lassa Fever Case Management. https://ncdc.gov.ng/themes/common/docs/protocols/92_1547068532.pdf.

[126] Mehand M.S., Al-Shorbaji F., Millett P., Murgue B. (2018). *The WHO R&D Blueprint: 2018* review of emerging infectious diseases requiring urgent research and development efforts. Antivir. Res..

[127] World Health Organisation (2023). Prioritizing Diseases for Research and Development in Emergency Contexts. https://www.who.int/activities/prioritizing-diseases-for-research-and-development-in-emergency-contexts.

[128] Bernasconi V., Kristiansen P.A., Whelan M., Román R.G., Bettis A., Yimer S.A., Gurry C., Andersen S.R., Yeskey D., Mandi H. (2020). Developing vaccines against epidemic-prone emerging infectious diseases. Bundesgesundheitsblatt Gesundheitsforschung Gesundheitsschutz.

[129] CEPI (2024). Testing the Tests: Scientists Seek Out Best on-the-Spot Diagnostics for Deadly Nipah and Lassa. CEPI Official Website. https://cepi.net//testing-tests-scientists-seek-out-best-spot-diagnostics-deadly-nipah-and-lassa.

[130] Epidemic Preparedness Innovations (2024). Enable Epi Study—Epidemic Preparedness Innovations. The Global Health Network. https://epi.tghn.org/epidemiology/epi-studies/.

[131] Goios A., Varma A., Kagia C., Otiende M., Suykerbuyk P. (2022). Enable Lassa Research Programme Mid-Term Workshop.

[132] The West Africa Lassa Fever Consortium (2023). A Clinical Development Strategy to Deliver New Lassa Fever Therapeutics. https://isaricdev.wpenginepowered.com/wp-content/uploads/2023/03/A-Clinical-Development-Strategy-to-Deliver-New-Lassa-Fever-Therapeutics.pdf.

[133] Mueller-Langer F. (2013). Neglected infectious diseases: Are push and pull incentive mechanisms suitable for promoting drug development research?. Health Econ. Policy Law.

[134] Garry R.F. (2023). Lassa fever—The road ahead. Nat. Rev. Microbiol..

[135] Melnik L.I. (2023). Lassa Virus Countermeasures. Curr. Top Microbiol. Immunol..

[136] Furuta Y., Komeno T., Nakamura T. (2017). Favipiravir (T-705), a broad spectrum inhibitor of viral RNA polymerase. Proc. Jpn. Acad. Ser. B.

[137] Oestereich L., Rieger T., Lüdtke A., Ruibal P., Wurr S., Pallasch E., Bockholt S., Krasemann S., Muñoz-Fontela C., Günther S. (2016). Efficacy of Favipiravir Alone and in Combination with Ribavirin in a Lethal, Immunocompetent Mouse Model of Lassa Fever. J. Infect. Dis..

[138] Rosenke K., Feldmann H., Westover J.B., Hanley P.W., Martellaro C., Feldmann F., Saturday G., Lovaglio J., Scott D.P., Furuta Y. (2018). Use of Favipiravir to Treat Lassa Virus Infection in Macaques. Emerg. Infect. Dis..

[139] Raabe V.N., Kann G., Ribner B.S., Morales A., Varkey J.B., Mehta A.K., Lyon G.M., Vanairsdale S., Faber K., Becker S. (2017). Favipiravir and Ribavirin Treatment of Epidemiologically Linked Cases of Lassa Fever. Clin. Infect. Dis..

[140] Cashman K.A., Wilkinson E.R., Posakony J., Madu I.G., Tarcha E.J., Lustig K.H., Korth M.J., Bedard K.M., Amberg S.M. (2022). Lassa antiviral LHF-535 protects guinea pigs from lethal challenge. Sci. Rep..

[141] Amberg S.M., Snyder B., Vliet-Gregg P.A., Tarcha E.J., Posakony J., Bedard K.M., Heald A.E. (2022). Safety and Pharmacokinetics of LHF-535, a Potential Treatment for Lassa Fever, in Healthy Adults. Antimicrob. Agents Chemother..

[142] Gowen B.B., Smee D.F., Wong M.-H., Pace A.M., Jung K.-H., Bailey K.W., Blatt L.M., Sidwell R.W. (2006). Combinatorial Ribavirin and Interferon Alfacon-1 Therapy of Acute Arenaviral Disease in Hamsters. Antivir. Chem. Chemother..

[143] Baize S., Marianneau P., Loth P., Reynard S., Journeaux A., Chevallier M., Tordo N., Deubel V., Contamin H. (2009). Early and Strong Immune Responses Are Associated with Control of Viral Replication and Recovery in Lassa Virus-Infected Cynomolgus Monkeys. J. Virol..

[144] Cross R.W., Heinrich M.L., Fenton K.A., Borisevich V., Agans K.N., Prasad A.N., Woolsey C., Deer D.J., Dobias N.S., Rowland M.M. (2023). A human monoclonal antibody combination rescues nonhuman primates from advanced disease caused by the major lineages of Lassa virus. Proc. Natl. Acad. Sci. USA.

[145] Cross R.W., Fenton K.A., Woolsey C., Prasad A.N., Borisevich V., Agans K.N., Deer D.J., Dobias N.S., Fears A.C., Heinrich M.L. (2024). Monoclonal antibody therapy protects nonhuman primates against mucosal exposure to Lassa virus. Cell Rep. Med..

[146] ALIMA (2024). First-Ever Global Alliance of Researchers, Health Workers, and Humanitarians Join Forces to Fight the Deadly Lassa Fever Virus. ALIMA—The Alliance for Medical Action. https://alima.ngo/en/press-releases/lassa-fever-alliance-virus/.

[147] Sulis G., Peebles A., Basta N.E. (2023). Lassa fever vaccine candidates: A scoping review of vaccine clinical trials. Trop. Med. Int. Health.

[148] Moore K.A., Ostrowsky J.T., Mehr A.J., Johnson A.R., Ulrich A.K., Moua N.M., Fay P.C., Hart P.J., Golding J.P., Benassi V. (2024). Lassa fever research priorities: Towards effective medical countermeasures by the end of the decade. Lancet Infect. Dis..

[149] Mateo M., Reynard S., Carnec X., Journeaux A., Baillet N., Schaeffer J., Picard C., Legras-Lachuer C., Allan R., Perthame E. (2019). Vaccines inducing immunity to Lassa virus glycoprotein and nucleoprotein protect macaques after a single shot. Sci. Transl. Med..

[150] Mateo M., Reynard S., Pietrosemoli N., Perthame E., Journeaux A., Noy K., Germain C., Carnec X., Picard C., Borges-Cardoso V. (2023). Rapid protection induced by a single-shot Lassa vaccine in male cynomolgus monkeys. Nat. Commun..

[151] Tschismarov R., Van Damme P., Germain C., De Coster I., Mateo M., Reynard S., Journeaux A., Tomberger Y., Withanage K., Haslwanter D. (2023). Immunogenicity, safety, and tolerability of a recombinant measles-vectored Lassa fever vaccine: A randomised, placebo-controlled, first-in-human trial. Lancet.

[152] ClinicalTrials (2020). No Study Results Posted|Safety, Tolerability and Immunogenicity of INO-4500 in Healthy Volunteers. ClinicalTrials.gov (US Government). https://clinicaltrials.gov/study/NCT03805984?tab=results.

[153] Marrero I. (2021). INO 4500, a DNA Based LASV Vaccine, Induces Robust T Cell Responses and Long-Term Memory Antigen-Specific T Cells|Vaccines Conferences|International Vaccines Congress. https://vaccinescongress.com/program/scientific-program/2021/ino-4500-a-dna-based-lasv-vaccine-induces-robust-t-cell-responses-and-long-term-memory-antigen-specific-t-cells.

[154] INOVIO Pharmaceuticals, Inc. (2022). INOVIO Provides an Update on Lassa Fever and MERS Programs. https://ir.inovio.com/news-releases/news-releases-details/2022/INOVIO-Provides-an-Update-on-Lassa-Fever-and-MERS-Programs/default.aspx.

[155] Baden M., Kieh L., Fitz-Patrick D., Diemert D., Mutua G. (2023). First Safety & Immunogenicity Data from a FIH, Placebo-Controlled, Dose-Escalation Trial of a Recombinant Vesicular Stomatitis Virus-Based Lassa Fever Vaccine in Healthy Adults. https://www.iavi.org/wp-content/uploads/2023/11/C102_ASTMH_Poster_Chicago2023.pdf.

[156] Abiola A. (2024). Time to Hope for a Lassa Fever Vaccine? GAVI, the Vaccine Alliance. https://www.gavi.org/vaccineswork/time-hope-lassa-fever-vaccine.

[157] Pan African Clinical Trials Registry (2021). A Phase 1 Randomized, Blinded, Placebo Controlled, Dose-Escalation and Dosing Regimen Selection Study to Evaluate the Safety and Immunogenicity of rVSV-Vectored Lassa Virus Vaccine in Healthy Adults at Multiple Sites in West Africa. Pan African Clinical Trials Registry. https://pactr.samrc.ac.za/TrialDisplay.aspx?TrialID=14618.

[158] Fahrni M.L., Ismail I.A.-N., Refi D.M., Almeman A., Yaakob N.C., Saman K.M., Mansor N.F., Noordin N., Babar Z.-U. (2022). Management of COVID-19 vaccines cold chain logistics: A scoping review. J. Pharm. Policy Pr..

[159] Leach M., Fairhead J. (2008). Understandings of immunization: Some west African perspectives. Bull World Health Organ..

[160] Agbede G.T., Emezirinwune D., Adedokun T., Idowu-Collins P. (2024). Vaccine Hesitancy in Nigeria: Overcoming Cultural, Linguistic and Religious Obstacles. Inf. Impact J. Inf. Knowl. Manag..

[161] Michael C.A., Ogbuanu I.U., Storms A.D., Ohuabunwo C.J., Corkum M., Ashenafi S., Achari P., Biya O., Nguku P., Mahoney F. (2014). An Assessment of the Reasons for Oral Poliovirus Vaccine Refusals in Northern Nigeria. J. Infect. Dis..

[162] World Health Organisation (2014). Report of the SAGE Working Group on Vaccine Hesitancy. https://www.asset-scienceinsociety.eu/sites/default/files/sage_working_group_revised_report_vaccine_hesitancy.pdf.

[163] Jones K.E., Patel N.G., Levy M.A., Storeygard A., Balk D., Gittleman J.L., Daszak P. (2008). Global trends in emerging infectious diseases. Nature.

[164] Cutler S.J., Fooks A.R., van der Poel W.H.M. (2010). Public Health Threat of New, Reemerging, and Neglected Zoonoses in the Industrialized World. Emerg. Infect. Dis..

[165] Pepin M., Tordo N. (2010). Emerging and re-emerging animal viruses. Foreword. Vet. Res..

[166] El Amri H., Boukharta M., Zakham F., Ennaji M.M. (2020). Emergence and Reemergence of Viral Zoonotic Diseases: Concepts and Factors of Emerging and Reemerging Globalization of Health Threats. Emerging and Reemerging Viral Pathogens.

[167] Baker R.E., Mahmud A.S., Miller I.F., Rajeev M., Rasambainarivo F., Rice B.L., Takahashi S., Tatem A.J., Wagner C.E., Wang L.-F. (2022). Infectious disease in an era of global change. Nat. Rev. Microbiol..

[168] Sinclair J. (2019). Importance of a One Health approach in advancing global health security and the Sustainable Development Goals. Rev. Sci. Tech. l’OIE.

[169] Cunningham A.A., Scoones I., Wood J.L.N. (2017). One Health for a changing world: New perspectives from Africa. Philos. Trans. R. Soc. B Biol. Sci..

[170] Abubakar I.R. (2017). Access to Sanitation Facilities among Nigerian Households: Determinants and Sustainability Implications. Sustainability.

[171] Yaya S., Hudani A., Udenigwe O., Shah V., Ekholuenetale M., Bishwajit G. (2018). Improving Water, Sanitation and Hygiene Practices, and Housing Quality to Prevent Diarrhea among Under-Five Children in Nigeria. Trop. Med. Infect. Dis..

[172] Akokuwebe M.E., Idemudia E.S. (2023). Fraud within the Nigerian health system, a double threat for resilience of a health system and the response to the COVID-19 pandemic: A review. Pan Afr. Med J..

[173] Kalbarczyk A., Davis W., Kalibala S., Geibel S., Yansaneh A., Martin N.A., Weiss E., Kerrigan D., Manabe Y.C. (2019). Research Capacity Strengthening in Sub-Saharan Africa: Recognizing the Importance of Local Partnerships in Designing and Disseminating HIV Implementation Science to Reach the 90–90–90 Goals. AIDS Behav..

